# A Case Report of Neonatal Vomiting due to Adrenal Hemorrhage, Abscess and Pseudohypoaldosteronism

**DOI:** 10.21980/J8QQ0B

**Published:** 2021-07-15

**Authors:** Raymen Rammy Assaf, Mary Jane Piroutek

**Affiliations:** * Harbor UCLA Medical Center, Department of Pediatric Emergency Medicine, Torrance, CA; ^ Children’s Hospital Orange County, Division of Emergency Medicine, Orange, CA

## Abstract

Neonatal adrenal hemorrhage is a rare phenomenon which may occur in utero or in the intrapartum period. This case illustrates ultrasound and subsequent abdominal computed tomography (CT) imaging of a 30-day-old neonate who initially presented with acute vomiting and was found to have an abscess at the site of a previous right-sided adrenal hemorrhage. Ultrasound-guided fine needle aspiration of the abscess was ultimately the key diagnostic and therapeutic intervention, which detected *Escherichia coli (E. coli)* infection. The discussion addresses the connection between key pathologic features of this case, while reviewing the acute management of clinical sequelae in the emergency department.

**Topics:**

Neonatal vomiting, adrenal hemorrhage, retroperitoneal abscess, pseudohypoaldosteronism.

**Figure f1-jetem-6-3-v13:**
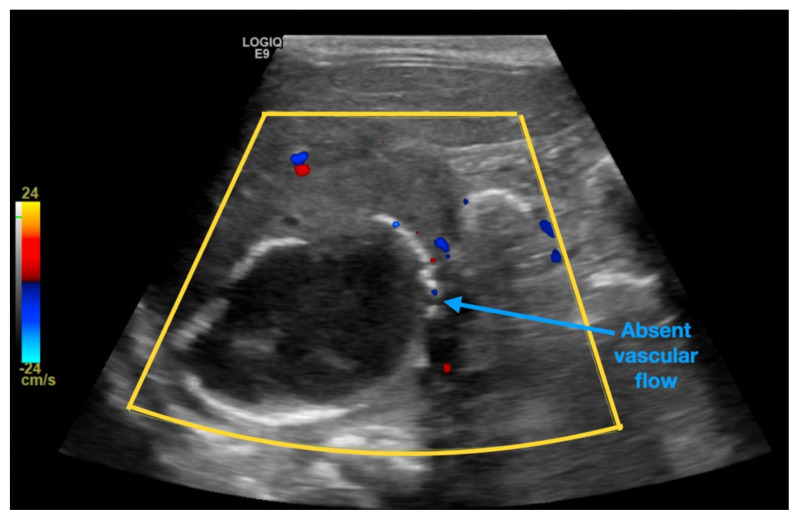


**Figure f2-jetem-6-3-v13:**
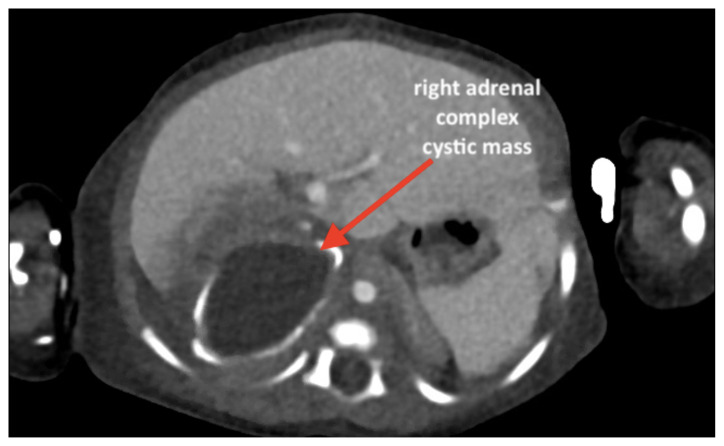


**Figure f3-jetem-6-3-v13:**
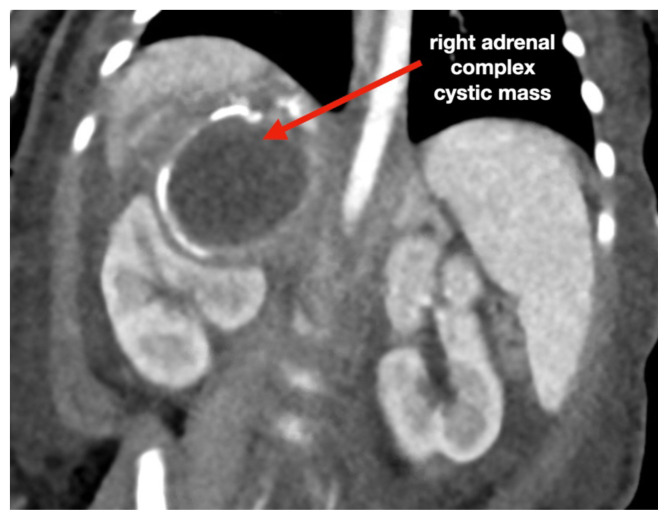


**Figure f4-jetem-6-3-v13:**
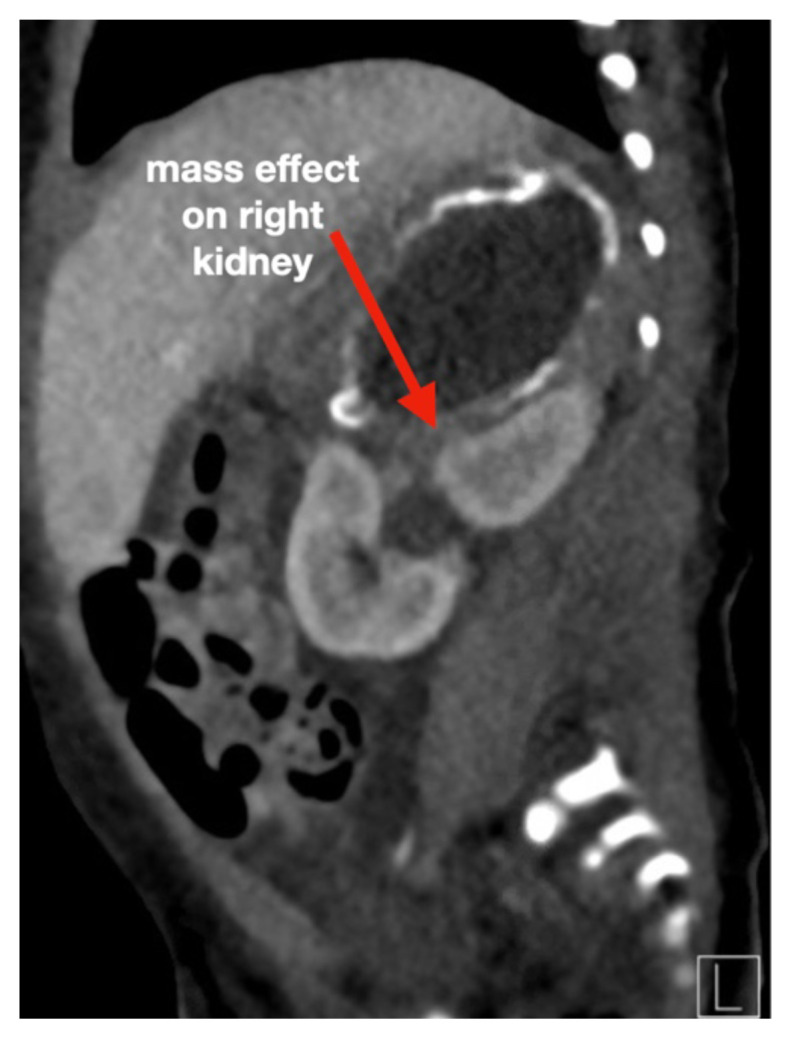


## Brief introduction

[Fig f1-jetem-6-3-v13][Fig f2-jetem-6-3-v13][Fig f3-jetem-6-3-v13][Fig f4-jetem-6-3-v13]Neonatal adrenal hemorrhage is a rare condition that affects less than 0.5% of newborns; it is thought to be due to the relatively large size and unique vascularity of the neonatal adrenal gland, particularly on the right side where it can be compressed between the liver and spine while undergoing acute venous pressure and/or hypoxic changes during labor.^1^,^2^ One theoretical complication involving the adrenal hemorrhage site is infection and bacterial seeding. Peri-nephric infection, urinary tract infection (UTI), and/or urinary tract malformation may contribute to the development of pseudohypoaldosteronism, which, especially in a neonate, may be misdiagnosed as congenital adrenal hyperplasia (CAH).^3^,^4^ This case report addresses an unusual cause of vomiting in a neonate presenting to the emergency department (ED), which was a retroperitoneal abscess found at the site of an old and previously undetected adrenal hemorrhage, complicated by profound electrolyte disturbances mimicking CAH. The featured ultrasound and CT images illustrate the dramatic adrenal lesion with evidence of chronicity. This case explores the most likely connection between the patient’s presentation and complications while reviewing the most relevant diagnostics and therapeutics involved.

## Presenting concerns and clinical findings

A previously healthy 30-day-old term male was brought to the emergency department (ED) by his mother for non-bilious emesis with each feeding, as well as decreased urine output for the past two days. He had made only four wet diapers in the last 48 hours and had become increasingly lethargic over the last 12 hours. No fever, difficulty breathing, diarrhea, or rashes were reported. The baby was exclusively breastfed but had had poor feeding with the onset of his symptoms. Birthweight was 3.66 kg and there were no reported complications at birth.

On physical exam, patient’s weight was 4.17 kg and he was afebrile, awake, in no respiratory distress, with a grade II/VI systolic ejection murmur, good pulses, and normal tone. His abdomen was soft and non-tender with no appreciable masses, testes descended bilaterally, and no edema.

Lab work demonstrated profound hyponatremia (serum sodium 111 mMol/L), mild hyperkalemia (potassium 6.2 mMol/L), moderate hypochloridemia (chloride 80 mMol/L), and mild acidemia (bicarbonate 20 mMol/L). Venous blood gas showed blood pH 7.37, CO2 35 mmHg, bicarbonate 19.5 mMol/L, and base deficit 5 mMol/L. Complete blood count showed a white blood cell count 12.4 K/UL, hemoglobin 13 gm/dL, hematocrit 37.3%, and platelet count 180 K/UL (manual differential: 32% neutrophils, 43% lymphocytes, 21% monocytes, 1% eosinophils, 3% band neutrophils). Erythrocyte sedimentation rate (ESR) and C-reactive protein (CRP) were not elevated. Urinalysis with microscopy via catheterized sample showed negative leukocyte esterase and nitrite, 10 WBC/HPF, 89 RBC/HPF, and moderate bacteria. Lumbar puncture results were unremarkable.

## Significant findings

An ultrasound (US) of the abdomen was obtained to evaluate for possible pyloric stenosis (see US transverse/dopper imaging). While imaging showed a normal pyloric channel, it revealed an unexpected finding: a complex cystic mass arising from the right adrenal gland (yellow outline), measuring 5.8 by 4.0 by 6.4 cm with calcifications peripherally and mass effect on the kidney without evidence of vascular flow (blue arrow). Computed tomography (CT) of the abdomen/pelvis with IV contrast was subsequently obtained and measured the lesion as 2.8 by 4.6 by 4 cm without evidence of additional masses, lymphadenopathy or left adrenal gland abnormality (see CT axial, coronal, and sagittal imaging).

## Patient course

In the ED, a normal saline bolus of 20 ml/kg was given for dehydration and a pediatric endocrinologist was consulted for management of suspected adrenal insufficiency, given the pattern of hyponatremia, hyperkalemia and metabolic acidosis. A stress dose of 25mg IV hydrocortisone was given and a D5NS drip initiated at maintenance rate with a goal to slowly increase serum sodium to 125 over 8 hours while the patient was kept NPO. Ceftriaxone 50 mg/kg was administered for UTI and the oncology service was consulted given the possibility of the adrenal mass representing a neuroblastoma. The patient was then admitted to the pediatric intensive care unit (PICU) for acute management of electrolyte derangement, further diagnostic workup, and sub-specialist evaluation.

Urine culture confirmed UTI, growing > 100,000 cfu/mL *Escherichia coli*. Steroid stress dosing, which was originally given to address adrenal insufficiency, was halted after two days because it did not appear to aid serum sodium correction. Further endocrinologic workup showed normal levels of serum cortisol, 17-hydroxyprogesterone, and dehydroepiandrosterone (DHEA), along with an appropriate response to a Cortrosyn stimulation test, making CAH and more generally adrenal insufficiency unlikely, despite the electrolyte imbalances detected in the ED. However, serum renin was found to be abnormally low, suggesting renal suppression (less than 0.167 ng/mL/hr, reference range 2–37 ng/mL/hr). An oncologic workup demonstrated negative urine catecholamine levels and chromogranin A.

Ultrasound-guided fine needle aspiration of the adrenal mass was performed by interventional radiology, with removal of 7 mL of blood-tinged thick green fluid. Pathology slides did not reveal malignant cells or fungal organisms; however, the specimen culture grew multiple colonies of *E. coli*. Pediatric surgery was consulted and recommended against surgical resection of the adrenal mass given there was no evidence of malignancy and the expected resolution of the lesion with aspiration and antibiotic therapy alone. The patient was discharged on daily in-home IV ceftriaxone, a total course of four weeks given his very young age and potential for recurrence or seeding of infection, and to assure complete resolution of the adrenal mass on surveillance imaging. He continues to feed, grow and develop normally on regular follow up, with resolution of the adrenal mass.

## Discussion

What is remarkable about this case is that despite suffering three rare but likely interrelated complications, each life-threatening, the patient made a complete recovery. *The first complication* was the undetected intrapartum right-sided adrenal hemorrhage, a condition that affects less than 0.5% of newborns as a complication of acute venous pressure and/or hypoxic changes during labor. *The subsequent complication* was abscess formation at the site of the resolving adrenal hemorrhage through bacterial seeding, via either urine or transient hematologic route - a phenomenon sparsely reported. *The final complication*, most likely following inflammatory changes caused by the abscess, was a profound hyponatremia with hyperkalemia and metabolic acidosis, all of which completely resolved with IV fluids and antibiotics, suggesting acquired (type 3) pseudohypoaldosteronism: a transient renal tubular resistance to aldosterone. While this electrolyte combination may be more readily attributed to CAH, especially in the ED, it can occur in infants as a rare complication of UTI or urinary tract malformation due to renal tubular inflammation and dysfunction.^3^,^4^ In this case, the adrenal abscess likely exerted mass effect on renal collecting tubules, interfering with aldosterone receptor and electrolyte channel activity. Given all three pathologic processes were ongoing at the time of this patient’s presentation to medical care, it is difficult to discern with certainty how each complication arose, but this proposed chronologic series appears to be the most likely.

Adrenal hemorrhage is not unique to neonates and may also occur in older age groups in the setting of trauma, coagulopathy, or existing neoplasm.^5^ Signs and symptoms on presentation are nonspecific, and often cases of non-traumatic adrenal hemorrhage are discovered incidentally while a patient is undergoing evaluation of another medical condition.^5^ Neonatal adrenal hemorrhage is associated with conditions leading to hemodynamic instability, including traumatic delivery, hypoxemia, sepsis, and coagulopathy.^6^ The condition may be discovered incidentally or may manifest as abdominal mass, scrotal hematoma or hypotension on exam, as well as unexplained anemia, jaundice, and/or abdominal calcification on lab and imaging workup.^7^ Ultrasonography may demonstrate a hypoechoic mass, an echogenic calcified mass, or a mass with mixed solid and liquid contents at the site of the adrenal gland and may be used serially to monitor for spontaneous resolution over a course of 90 days, during which the lesion undergoes cystic changes.^8^ Lesions with similar appearance and anatomic location on ultrasonography include neuroblastoma, Wilms tumor, adrenal abscess, CAH, adrenal or splenic cyst, and lymphangioma.^9^

Historically, it has been hypothesized that adrenal hemorrhage is a likely trigger for adrenal abscess in the newborn period.^10^ Neonatal adrenal abscess is an extremely rare condition which may present with fever, irritability, vomiting, and/or abdominal distention.^11^ Although scantly described in the literature, imaging modalities most often include abdominal US and CT, and in rare cases they may present bilaterally.^11^–^13^ US may demonstrate a cystic mass with internal heterogeneous echoes, while contrast-enhanced CT abdomen may show loculated fluid with rim enhancement and renal mass effect in the suprarenal region.^13^ Due to mass effect and proximity to the kidneys, perinephric infection, UTI, or urinary tract malformation may contribute to the development of pseudohypoaldosteronism, which, especially in a neonate, may be misdiagnosed as CAH. While adrenal hemorrhage often spontaneously resolves with conservative management, the presence of an abscess is associated with high mortality rates and requires prolonged antibiotic administration and drainage with minimally invasive technique such as with ultrasound-guided percutaneous fine needle aspiration.^12^ Follow up course is typically uneventful with no reported cases of recurrence.^10^–^13^

This case describes a neonatal adrenal hemorrhage complicated by the development of a retroperitoneal abscess and secondary electrolyte disturbance. These are rare but serious events requiring medical and procedural intervention. Abdominal US and contrast-enhanced CT remain the imaging modalities of choice.^13^ Adrenal hemorrhage and abscess both present with non-specific symptoms and may even be discovered incidentally while performing medical workup for another cause of neonatal emesis. Clinical presentation may mimic that of CAH and may lead to significant morbidity or mortality if not timely addressed; however, this case demonstrates an excellent outcome with early supportive care and medical management.

## Supplementary Information


















