# Supplementary figures and images for: A Case Report of Neonatal Vomiting due to Adrenal Hemorrhage, Abscess and Pseudohypoaldosteronism
Source: J Educ Teach Emerg Med. 2021 Jul 15;6(3):V13–7. doi: 10.21980/J8QQ0B (PMC10332690; doi:10.21980/J8QQ0B)

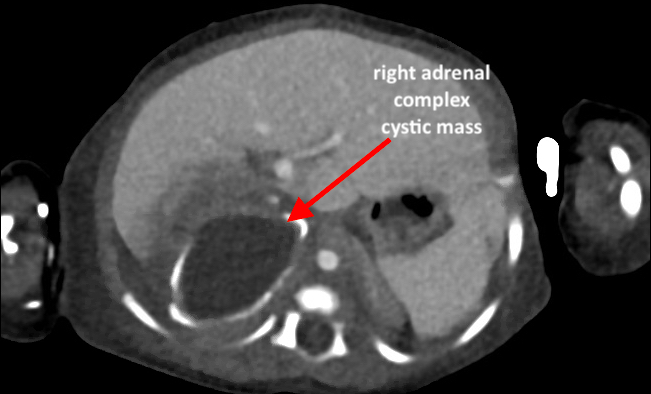

Supplement: Supplementary file 1 [file jetem-6-3-v13-supp1.jpg]

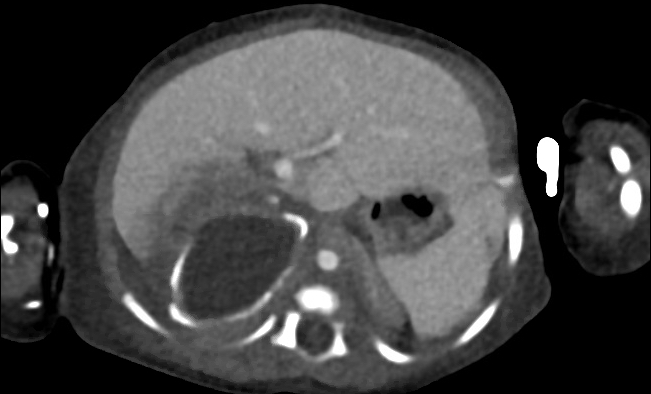

Supplement: Supplementary file 2 [file jetem-6-3-v13-supp2.jpg]

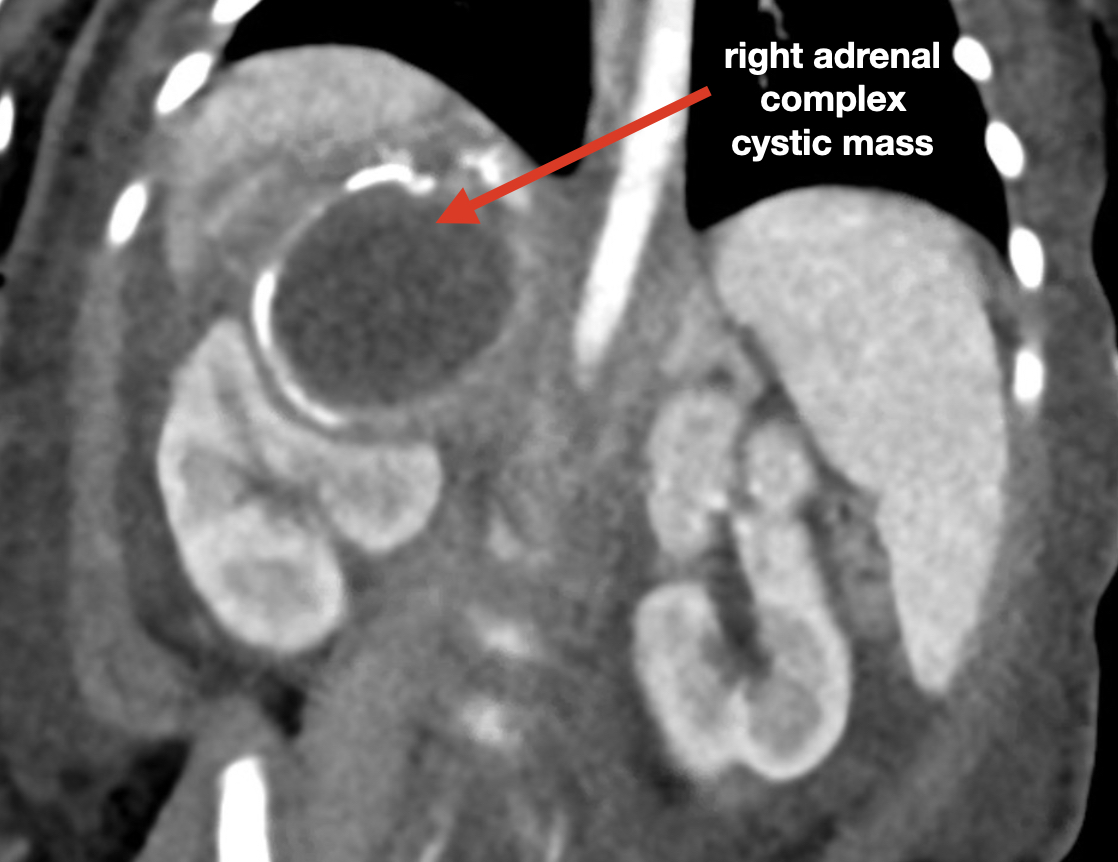

Supplement: Supplementary file 3 [file jetem-6-3-v13-supp3.jpg]

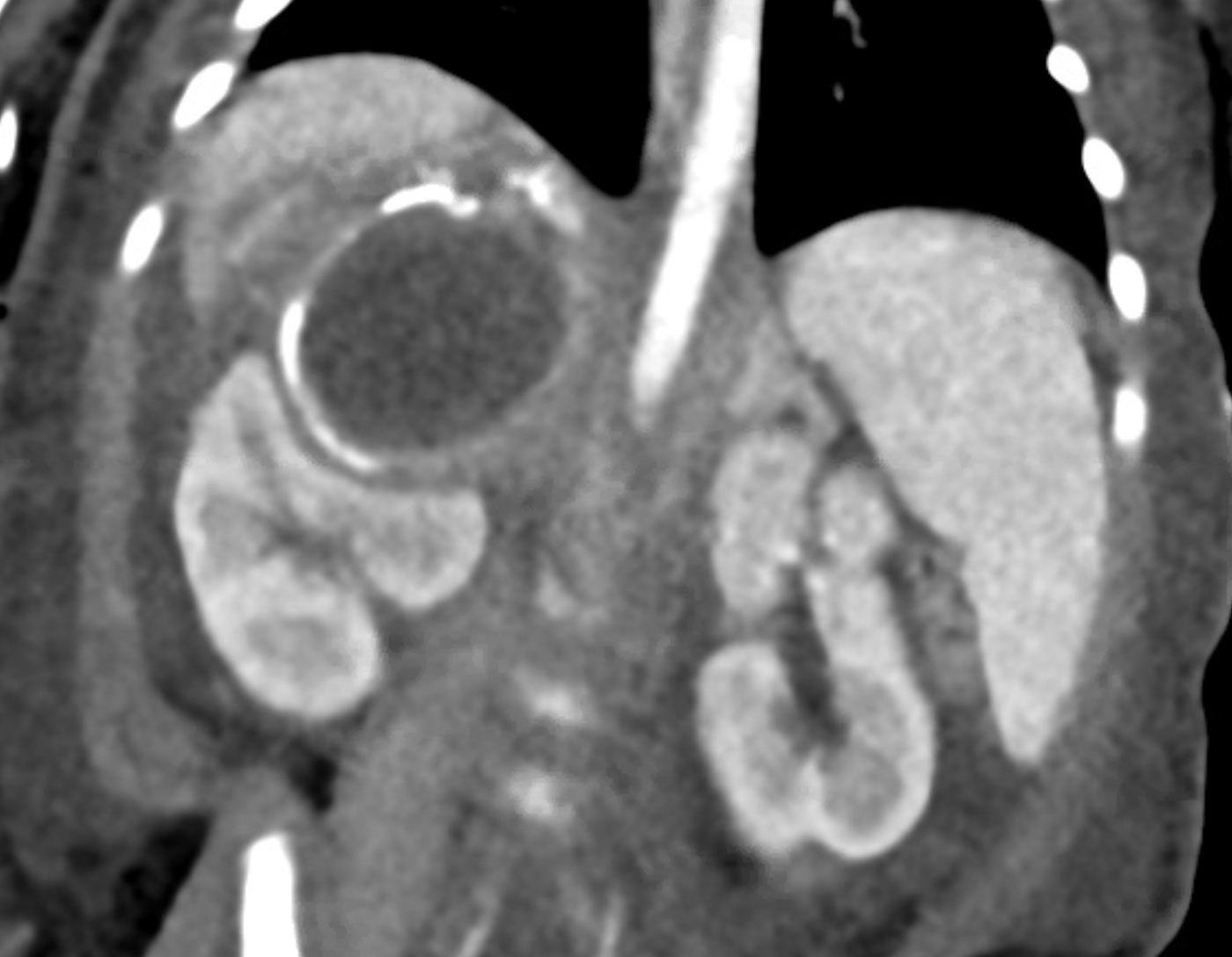

Supplement: Supplementary file 4 [file jetem-6-3-v13-supp4.jpg]

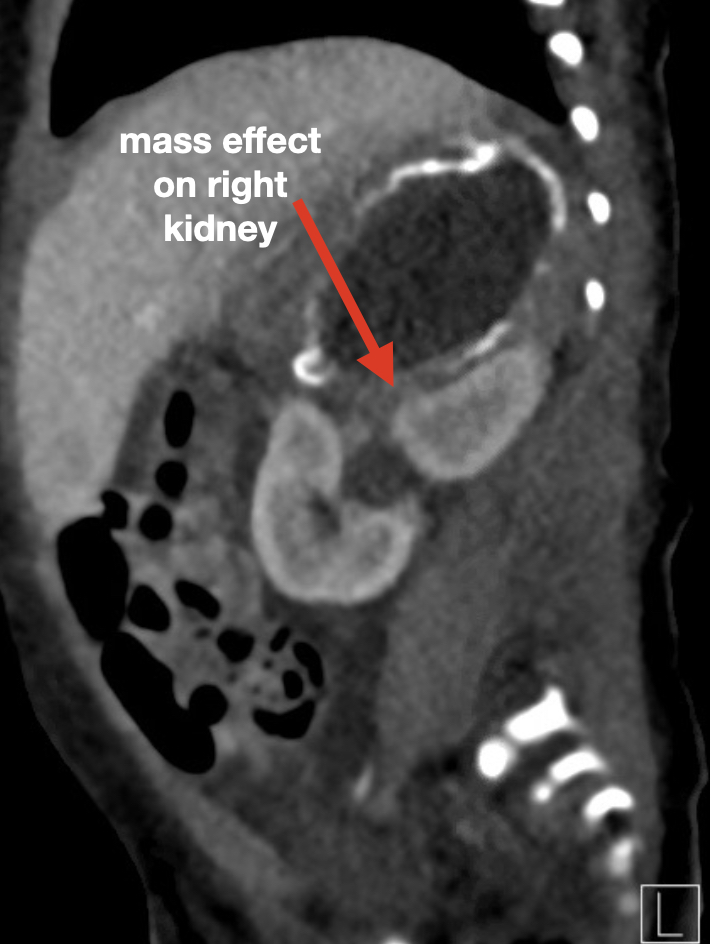

Supplement: Supplementary file 5 [file jetem-6-3-v13-supp5.jpg]

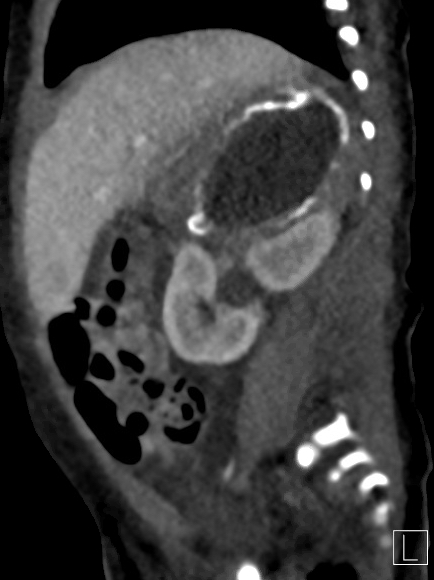

Supplement: Supplementary file 6 [file jetem-6-3-v13-supp6.jpg]

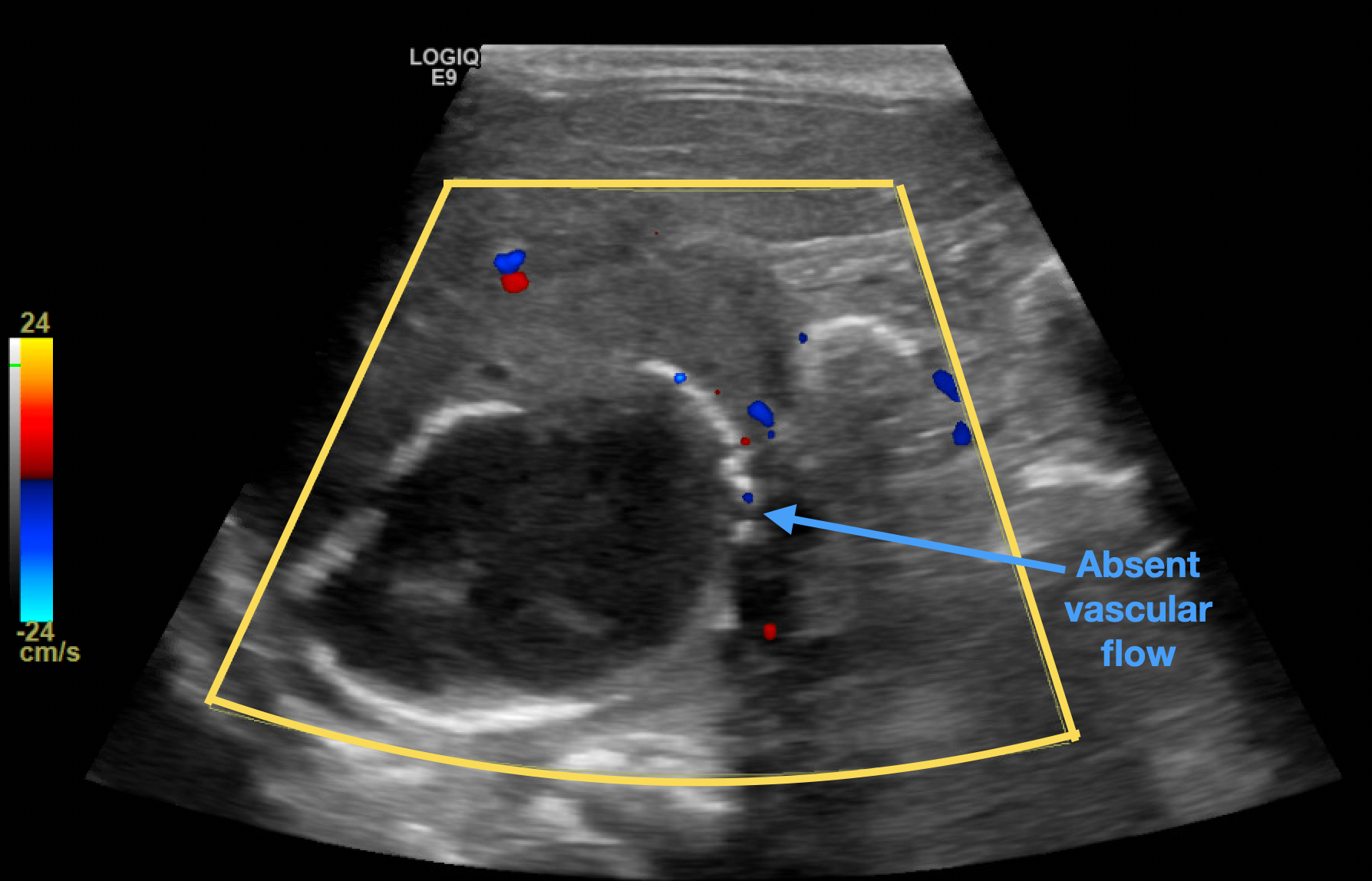

Supplement: Supplementary file 7 [file jetem-6-3-v13-supp7.jpg]

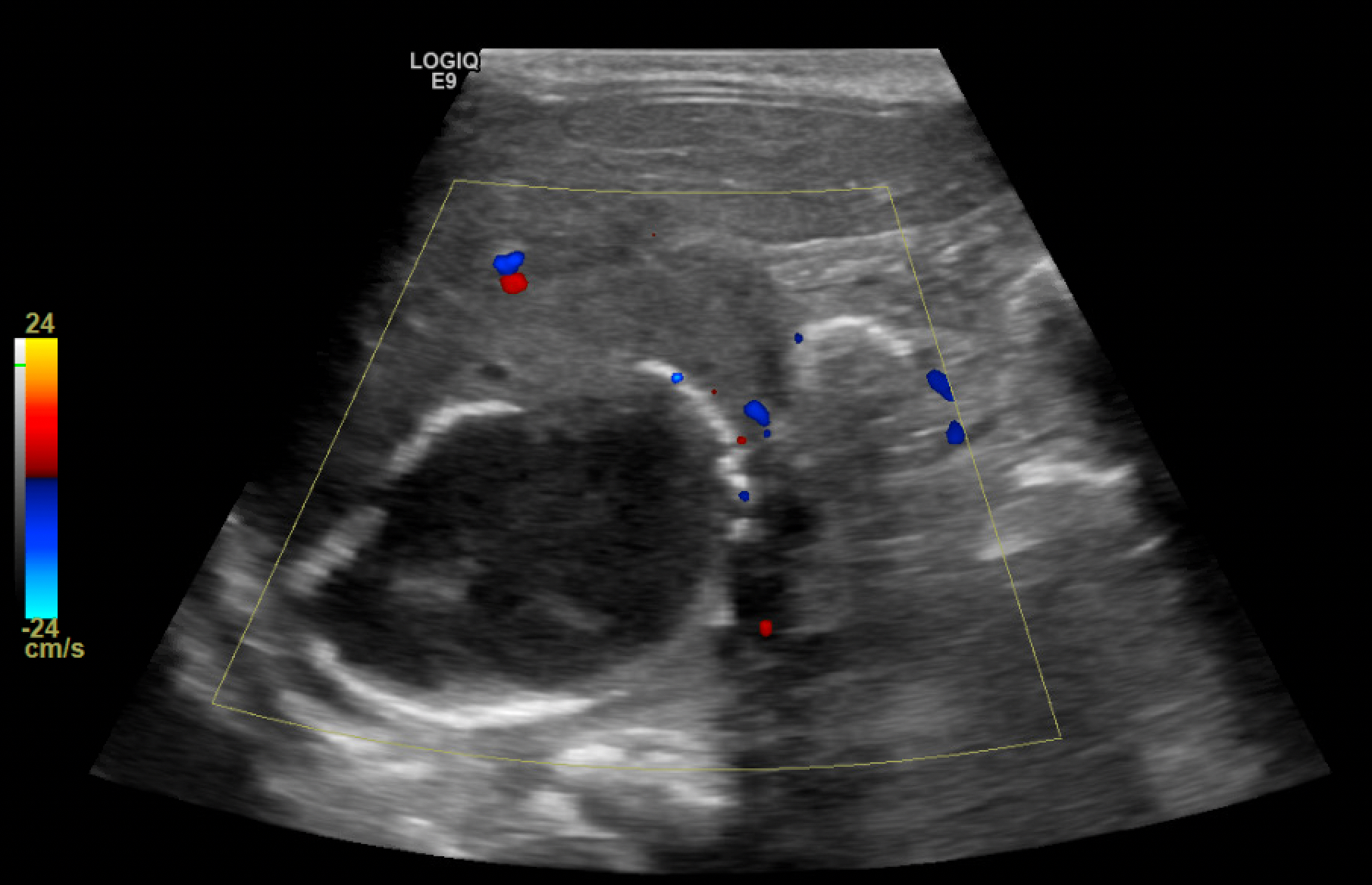

Supplement: Supplementary file 8 [file jetem-6-3-v13-supp8.jpg]

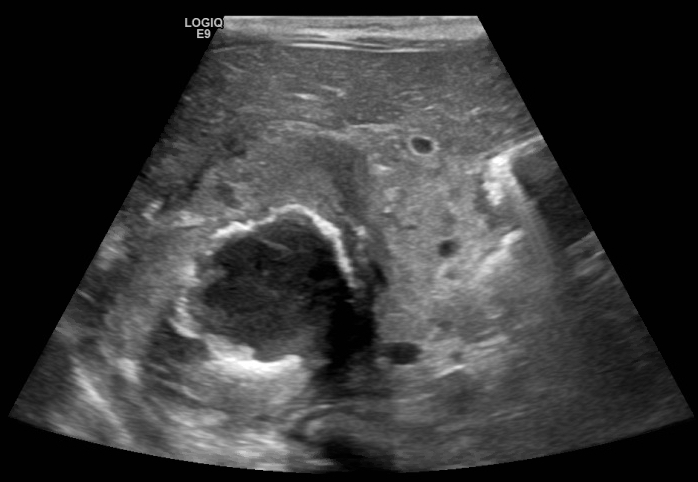

Supplement: Supplementary file 9 [file jetem-6-3-v13-supp9.jpg]
